# Baseline Micronuclei Frequency in Children: Estimates from Meta- and Pooled Analyses

**DOI:** 10.1289/ehp.7806

**Published:** 2005-05-31

**Authors:** Monica Neri, Marcello Ceppi, Lisbeth E. Knudsen, Domenico Franco Merlo, Roberto Barale, Riccardo Puntoni, Stefano Bonassi

**Affiliations:** 1 Unit of Epidemiology and Biostatistics, National Cancer Research Institute, Genoa, Italy; 2 Institute of Public Health, University of Copenhagen, Copenhagen, Denmark; 3 Unit of Molecular Epidemiology, National Cancer Research Institute, Genoa, Italy; 4 Dipartimento di Scienze dell’Uomo e dell’Ambiente, University of Pisa, Pisa, Italy

**Keywords:** biomarker, children, environmental exposure, genetic damage, meta-analysis, micronucleus assay, molecular epidemiology, pooled analysis

## Abstract

The number of studies evaluating the effect of environmental exposure to genotoxic agents in children has rapidly increased in the last few years. The frequency of micronuclei (MN) in peripheral blood lymphocytes determined with the cytokinesis block assay is among the most popular biomarkers used for this purpose, although large inter- and intralaboratory variability of this end point has been observed in population studies. The availability of reference measures is therefore necessary for laboratories to validate protocols and analytical procedures, and for molecular epidemiologists, as well, to estimate the statistical power of studies and to assess the quality of data. In this article, we provide estimates of the baseline frequency of MN in children, conducting a meta-analysis of MN frequency reported by field studies in children and a pooled analysis of individual data [available from published studies and from the Human Micronucleus International Collaborative Study (HUMN) database]. Thirteen articles were selected for meta-analysis, and individual data included in the pooled analysis were retrieved from the databases of 12 laboratories. Overall means of 4.48 [95% confidence interval (CI), 3.35–5.98] and 5.70 (95% CI, 4.29–7.56) MN per 1,000 binucleated cells were estimated by the meta- and pooled analysis, respectively. A clear effect of age was detected, even within the restricted range of pediatric age considered, with significantly lower frequency values in newborns. No influence of sex was found. The study showed the advantage of using data from large collaborative studies and suggested a synergistic use of meta- and pooled analysis.

The number of studies evaluating the effect of environmental exposure to genotoxic agents in children has rapidly grown in the last years (Neri et al. 2005b; Suk et al. 2003), boosted by two main considerations: *a*) Children may be more sensitive than adults to genotoxic agents, and *b*) genetic damage occurring at young ages may affect the lifetime risk of delayed adverse health outcomes (Landrigan et al. 2003; Wild and Kleinjans 2003).

Among the several adverse health effects that have been studied in children exposed to environmental hazards, genetic damage has received a particular interest, especially after the recent publication of epidemiologic studies showing that a high frequency of chromosome damage predicts cancer in healthy adults (Bonassi et al. 2004).

The use of genetic biomarkers in children raises a number of issues (Neri et al. 2005a). On the other hand, studies in children are essential to make use of the findings in public and environmental health. Ethical issues are related to protection of privacy, causing no harm, and leaving the child with a “feel-good experience.” Legal issues of data protection, confidentiality, and autonomy of the child are also important. Further, a direct application to pediatric populations of biomarkers of genetic damage that have been proven useful in adults may be misleading. Differences in exposure to and intake of environmental agents, xenobiotic metabolism, and the role of infectious diseases may alter the reliability of some biomarkers when translated from adults to children. Finally, to allow a correct interpretation of findings from these studies, a basic question must be addressed: What is the estimate of the spontaneous occurrence of genetic damage in children?

The micronucleus (MN) test in peripheral blood lymphocytes with the cytokinesis block method is one of the most popular assays of genetic damage in human biomonitoring (Bonassi et al. 2005). Details about the assay can be found in the articles published by the Human Micronucleus International Collaborative Study (HUMN) (Fenech et al. 1999, 2003). The growing interest in this test, mostly due to the easy use of MN in monitoring exposure to genotoxic agents, is fueled also by the accumulating evidence that the frequency of MN in healthy subjects may be considered a marker of risk for cancer (Tucker and Preston 1996) and cardiovascular disease (Andreassi and Botto 2003). The performance of this biomarker in field studies involving children exposed to environmental agents has been recently reviewed (Neri et al. 2003, 2005b).

Despite the large number of advantages that justify the popularity of this assay, there is an evident limitation—the large inter- and intralaboratory variability in the MN frequency. This variability may be explained partially by technical reasons, genetic variability, or sampling error. However, interscorer discrepancy and protocol differences (when different laboratories are involved) have been shown to be the most important sources of variability (Bonassi et al. 2001).

The meta- and pooled analyses represent the ideal statistical tools for computing summary estimates of a biomarker frequency using data from different studies (Greenland 1987). The advantages and the limitations of meta- and pooled analysis have been discussed in many articles, and in many aspects these methods are complementary (Taioli and Bonassi 2002).

In this study, we identified the published studies reporting MN during childhood (age range, 0–18 years) with the aim of performing a meta-analysis of MN frequency for referent children and providing a meta-estimate of the MN baseline value. Moreover, a pooled analysis of individual data available from published studies and from the HUMN database (Bonassi et al. 2001) was performed with the same purpose.

## Materials and Methods

### Search strategy and studies selection.

Individuals from birth to late adolescence (age range, 0–18 years) were considered as children. The studies selected for inclusion in the meta-analysis were identified by systematically searching the MedLine/PubMed database (National Library of Medicine, National Institutes of Health, Bethesda, MD, USA; http://www.ncbi.nlm.nih.gov/PubMed). The terms “micronucleus tests” existing since 1989 and “micronuclei” existing since 1990 were used as medical subject heading (MeSH) key word. To cover previous years, a free text search with terms “micronucleus” and “micronuclei” was performed. The categories “All Child: 0–18 years,” “Human,” “Language: English,” and “Publication Date: from January 1st, 1985 to September 1st, 2004” were set as search limits. This allowed retrieving 168 citations.

Only studies measuring MN frequency in lymphocytes with the cytokinesis block method (Fenech and Morley 1985) and with at least 10 subjects in the referent group were included. Referent children exposed to genotoxic agents or affected by any disease were also excluded from the statistical analyses. MN frequency was always reported as the number of MN per 1,000 (‰) binucleated cells. According to these criteria, 13 studies (Table 1), accounting for 440 subjects, were found to be suitable for the meta-analysis (Barale et al. 1998; Bilban and Vaupotic 2001; Da Cruz et al. 1994; Dulout et al. 1996; Fellay-Reynier et al. 2000; Fenech et al. 1997; Livingston et al. 1997; Maluf and Erdtmann 2001; Migliore et al. 1991; Mikhalevich et al. 2000; Shi et al. 2000; Vleminckx et al. 1997; Zotti-Martelli et al. 1999). One study with a small number of 19-year-old subjects was also included (Barale et al. 1998). Three different referent groups identified in a single study (Vleminckx et al. 1997) were treated as independent in the analysis.

Six of the 13 studies included in the meta-analysis reported individual data for 103 children (da Cruz et al. 1994; Dulout et al. 1996; Fellay-Reynier et al. 2000; Maluf and Erdtmann 2001; Migliore et al. 1991; Mikhalevich et al. 2000) and were used also in the pooled analysis, together with 229 subjects from the HUMN database (Bonassi et al. 2001).

### Statistical methods.

#### Meta-analysis.

We computed a summary estimate of the MN frequency applying the random-effects linear model to the natural logarithm of the MN mean of each study (DerSimonian and Laird 1986). This model takes into account two sources of variability: the error in estimating the *i*th MN mean in repeated samples from the same population the *i*th study belongs to, and the heterogeneity between studies. Although the former variability is assumed to be known and estimated by the standard error reported for each study, the latter (heterogeneity) was estimated from the model and found to be highly significant [*Q* = 308.14 (14 df), *p* < 0.001]. The analysis was carried out with STATA statistical software (STATA statistical software, release 7.0; Stata Corp., College Station, TX, USA); the random-effects model was performed with the procedure META, and the diagnostics, such as funnel plot, with the procedures METAINF and METABIAS.

#### Pooled analysis.

We obtained sex-specific and age-group–specific MN mean frequencies by fitting a negative binomial random-effects model to the MN count of each subject (Lindsey 1995). The negative binomial model was used to account for data overdispersion, whereas the clustered nature of data (i.e., the fact that the correlation between data within laboratories is likely to be larger than that between laboratories) was taken into consideration by introducing in the model a random effect due to the laboratory. The analysis was performed with MLwiN statistical software (version 1.10; Centre for Multilevel Modelling, Institute of Education, University of London, London, UK).

## Results

### Meta-analysis.

The MN frequency for each study included in the meta-analysis is reported in Table 1 along with the size of the study and the age range investigated. The overall meta-estimate of the MN frequency was 4.48 ‰ [95% confidence interval (CI), 3.35–5.98]. To evaluate the impact of single studies on this summary estimate of MN frequency, we performed a sensitivity analysis estimating the overall MN frequency after cyclically removing single studies. This approach showed the absence of influential studies, with meta-estimates of the MN frequency ranging from 4.77 to 4.22 ‰.

### Pooled analysis.

The layout of data selected for the pooled analysis is reported in Table 2. This analysis is more powerful with respect to meta-analysis because potential confounding factors, such as age and sex, are accounted for. Age-group–specific and sex-specific pooled estimates computed from the random-effects model are shown in Table 3. Poor information was available about ethnic characteristics, although the large majority of children in the study came from European countries. The pooled mean estimate of the MN frequency was 5.70 ‰ (95% CI, 4.29–7.56) when all 332 referent subjects were considered. While pooled-mean values were similar for males (5.94 ‰; 95% CI, 4.39–8.04) and females (5.54 ‰; 95% CI, 4.13–7.4), MN frequency was clearly associated with age. Frequency values were very low in children < 1 year of age (3.27 ‰; 95% CI, 2.22–4.82) and increased significantly thereafter, reaching the level of 7.05 ‰ (95% CI, 5.01–9.94) in the 15- to 19-year-old age group (chi-square test for trend, *p* < 0.001).

## Discussion

The main purpose of this study was to provide reference values for researchers planning studies on genomic damage in children. We used two complementary approaches to compute summary measures of baseline MN frequency in children. Meta-analysis provided summary measures that, although affected by a certain degree of heterogeneity, are considered to be representative of studies published in the literature. Pooled analysis, although limited to six published studies (those for which individual data were available), included 229 subjects (from six laboratories) from the HUMN database (Bonassi et al. 2001) and allowed the computation of pooled estimates adjusted for age and sex.

The estimates of the baseline MN frequency in children obtained by the two approaches were by large consistent, especially considering CIs. The lower mean value estimated by meta-analysis (4.48 vs. 5.70 ‰, adjusted for age and sex), considering that mean ages in the laboratories contributing data to meta-analysis and in those considered for the pooled analysis were similar, is likely to be attributable to a different distribution of absolute values of MN frequency in the two sets of data. This is not surprising because studies based on cytogenetic biomarkers suffer from a certain degree of heterogeneity among laboratories, and absolute values may differ even largely.

The availability of reference values is important for research teams and laboratories that need to validate protocols and analytical procedures as well as to estimate the statistical power of field studies and check the quality of data. For this purpose, baseline MN frequencies for the cytokinesis block assay have been published for an adult population (Bonassi et al. 2001).

Age is the most important predictor of MN frequency, as described by cooperative studies and reported in the literature (Bolognesi et al. 1997; Bonassi et al. 2001). However, despite the high number of subjects evaluated, only differences between the extent of genetic stability in adults and in children were described. Recently, data from a review of studies conducted in children exposed to a variety of mutagens, although limited, pointed to an influence of age even in the first two decades of life (Neri et al. 2003).

In the present study, the effect of age was evident even within the restricted age range considered in pooled analysis (0–19 years). The low frequency of MN detected in children < 1 year of age is noteworthy, given the growing number of studies performed on the cord blood, an easily accessible source of DNA. MN numbers were very low at birth (3.27 ‰) and increased by 66% in children 1–4 years of age (5.43 ‰), an increase that accounted for most of the age effect on MN frequency described in the literature. Besides the major challenges posed by the leaving of the protected intrauterine environment, other changes occurring in the first years of age, such as solid diet, vaccinations, and viral diseases, provide plausible explanations for this dramatic increase.

No difference in MN frequency was found by sex in our pooled analysis. The effect of sex has been repeatedly reported in adult populations, and a recently published pooled analysis estimated a 19% higher MN frequency in females than in males (Bonassi et al. 1995, 2001). However, the influence of sex was limited (and not statistically significant) in subjects ≤40 years of age and became more pronounced in older subjects (Bonassi et al. 2001). Possible biologic explanation include a sex-related aneuploidy phenomenon and the implication of sexual hormones (Bonassi et al. 1995). Findings reported by Neri et al. (2003) failed to show any clear effect of sex on MN frequency in children.

In conclusion, these results address an increasing request from researchers performing epidemiologic studies in children based on biomarkers—specifically, the availability of specific reference values in pediatric populations. The MN baseline values provided here are meant for the planning phase of a study and should not justify the conduct of future studies among children that exclude the identification of properly selected referent (control) subjects. Besides the main study findings, we showed here the advantage of using data from large collaborative research projects to improve the design of future field studies, including the efficiency of the statistical analysis.

## Figures and Tables

**Table 1:** Field studies of MN in children included in the meta-analysis.

Reference	No. of referent children (total no. of subjects in study)	MN (mean ± SD)	Age range (years)
Shi et al. 2000	20 (68)	1.70 ± 1.83	0–10
Barale et al. 1998	136 (1,650)	2.20 ± 2.22	0–19
Zotti-Martelli et al. 1999	30 (72)	2.26 ± 2.35	15 ± 2.0^a^
Fellay-Reynier et al. 2000	20 (41)	2.71 ± 2.60	0–18
Vleminckx et al. 1997^b^	33 (220)	2.94 ± 2.46	6–15
	31 (220)	4.19 ± 3.50	6–14
	25 (220)	4.76 ± 5.00	6–15
Migliore et al. 1991	15 (45)	4.14 ± 1.75	1–12
Maluf and Erdtmann 2001	30 (74)	4.65 ± 2.25	0–17
Livingston et al. 1997	31 (80)	5.16 ± 2.51	4–14
Dulout et al. 1996	12 (44)	5.58 ± 5.51	8–14
Da Cruz et al. 1994	16 (276)	7.33 ± 3.88	1–18
Bilban and Vaupotic 2001	20 (105)	9.00 ± 3.80	8–12
Fenech et al. 1997	11 (116)	9.80 ± 3.32	12–15
Mikhalevich et al. 2000	10 (30)	9.92 ± 2.70	14–17
Meta-estimate	440 (3,261)	4.48 ± 0.66^c^	0–19

a Mean ± SD.

b Three independently selected groups of referent children were included in the study (see “Materials and Methods” for details).

c SE approximate.

**Table 2:** Field studies of MN in children included in the pooled analysis and crude pooled estimates.

Author	No. of referents	MN (mean ± SD)	Range	Age range (years)	Males (%)
Barale (HUMN)	119	2.43 ± 2.39	0.00–10.07	9–18	56
Fellay-Reynier et al. 2000	20	2.71 ± 2.60	0.00–6.78	0–18	30
Migliore et al. 1991	15	4.14 ± 1.75	1.88–7.53	1–12	40
Maluf and Erdtmann 2001	30	4.65 ± 2.24	1.50–12.00	0–17	60
Dulout et al. 1996	12	5.58 ± 5.47	0.00–19.00	8–14	25
Garcia (HUMN)	19	5.63 ± 3.32	0.00–10.00	7–15	32
Da Cruz et al. 1994	16	7.33 ± 3.88	1.00–18.00	1–18	44
Vorobtsova (HUMN)	8	7.38 ± 3.07	2.00–12.00	9–17	38
Chang et al. (HUMN)	54	8.54 ± 7.71	1.12–49.30	0–17	57
Scarfì et al. (HUMN)	22	9.44 ± 3.89	3.68–19.19	0–18	45
Mikhalevich et al. 2000	10	9.92 ± 2.70	5.77–14.00	14–17	40
Muller et al. (HUMN)	7	10.29 ± 9.76	3.00–31.00	7–14	14
Crude pooled estimate	332	5.23 ± 5.07	0.00–49.30	0–18	49

HUMN, data from the HUMN database (Bonassi et al. 2001).

**Table 3:** Pooled analysis: estimated mean values of MN by sex and age group (random-effects model) and pooled estimates adjusted by sex and age.

	Females		Males		Pooled	
Age (years)	No.	Estimated mean (95% CI)	No.	Estimated mean (95% CI)	No.	Estimated mean (95% CI)
< 1	25	3.21 (2.17–4.76)	26	3.40 (2.26–5.10)	51	3.27 (2.22–4.82)
1–4	13	5.34 (3.41–8.36)	8	5.64 (3.56–8.96)	21	5.43 (3.48–8.48)
5–9	27	5.47 (3.82–7.82)	22	5.78 (4.03–8.29)	49	5.62 (3.97–7.96)
10–14	54	5.88 (4.23–8.16)	52	6.21 (4.44–8.68)	106	6.02 (4.37–8.30)
15–18	51	6.90 (4.86–9.82)	54	7.30 (5.11–10.4)	105	7.05 (5.01–9.94)
Pooled	170	5.54 (4.13–7.4)	162	5.94 (4.39–8.04)	332	5.70 (4.29–7.56)
